# A Multiconstrained Grid Scheduling Algorithm with Load Balancing and Fault Tolerance

**DOI:** 10.1155/2015/349576

**Published:** 2015-06-03

**Authors:** P. Keerthika, P. Suresh

**Affiliations:** ^1^Department on Computer Science and Engineering, Kongu Engineering College, Perundurai, Erode, Tamilnadu 638052, India; ^2^Department on Information Technology, Kongu Engineering College, Perundurai, Erode, Tamilnadu 638052, India

## Abstract

Grid environment consists of millions of dynamic and heterogeneous resources. A grid environment which deals with computing resources is computational grid and is meant for applications that involve larger computations. A scheduling algorithm is said to be efficient if and only if it performs better resource allocation even in case of resource failure. Allocation of resources is a tedious issue since it has to consider several requirements such as system load, processing cost and time, user's deadline, and resource failure. This work attempts to design a resource allocation algorithm which is budget constrained and also targets load balancing, fault tolerance, and user satisfaction by considering the above requirements. The proposed Multiconstrained Load Balancing Fault Tolerant algorithm (MLFT) reduces the schedule makespan, schedule cost, and task failure rate and improves resource utilization. The proposed MLFT algorithm is evaluated using Gridsim toolkit and the results are compared with the recent algorithms which separately concentrate on all these factors. The comparison results ensure that the proposed algorithm works better than its counterparts.

## 1. Introduction and Related Works

The computational power of individual computers is rapidly increasing from time to time. For problem solving in the fields like earth system sciences, financial modeling, and high energy physics, the approaches involving computation are widely used. But for these applications, the computational power of a single computer is not sufficient. It has limited resources and is not suitable for computation-intensive applications. In order to meet the computational demand, powerful distributed and parallel systems with more number of processors are developed. But few applications like parameter search problems need more number of resources which led to a solution of collecting and utilizing distributed resources owned by different institutions and domains. This distributed computing infrastructure is called grid.

Based on functionality, grid can be classified as computational grid and data grid. The resources involved in computational grids are computational resources such as processors. It is mainly used for computation intensive applications and data intensive applications. The applications which requires more time for computation are termed as computation intensive applications and the applications which require more time for data retrieval than computation are termed as data intensive applications. In data grid, the resources are storage resources like memory and mainly deal with data storage.

A grid system comprises a scheduler, grid portal, and a Grid Information Service (GIS). The scheduler or the grid broker is responsible for mapping of tasks to their suitable resources. It allows the users to request for resource allocation. This process is termed as scheduling. Scheduling can be varied as static scheduling and dynamic scheduling. The users communicates with the scheduler through grid portal. They have several Quality of Service (QoS) requirements of their task towards execution. The QoS requirements can be based on processing power, operating system, architecture, deadline, cost of execution, and bandwidth. Apart from scheduling, a grid must ensure many aspects such as balanced load of resources, failure handling mechanisms, security of data, and user satisfaction. These several independent issues make grid scheduling as a NP-complete problem [1].

The schedulers can be deployed level by level. The local scheduler is deployed within a cluster and is responsible for scheduling within the cluster. The scheduler at the top level is the grid broker. Scheduling can be centralized, decentralized, and hierarchical. In centralized scheduling, the scheduler has more control over the resources. In decentralized scheduling, there is no central entity to have control over the resources and the scheduling decisions are made individually. In hierarchical scheduling, different levels of schedulers are deployed and scheduling is done at all the levels.

The proposed algorithm suits for computational grids with computing resources and scheduling is done by concentrating on load balancing, fault tolerance, and several QoS requirements such as budget or cost and user deadline. The remaining part of this paper is organized with materials and methods which explain the works done previously with these factors and the newly proposed algorithm's architecture and nature. Then the experimental results are shown with comparisons and conclusions.

The grid computing environment comprises heterogeneous resources which are distributed geographically. Hence, identification of a suitable resource for the submitted task is a tedious process. Many researchers have proposed algorithms for mapping of tasks to resources. Some of them concentrate on user satisfaction, some on load balancing, and some on fault tolerance.

An algorithm is proposed in [1] that begins with Min-min algorithm if the number of available resources is odd and starts with Max-min algorithm if the number of available resources is even. The remaining tasks are assigned to their appropriate resources by one of the two strategies, alternatively.

A minimum time to release scheduling algorithm [2] has been discussed which depends on the time to release (TTR). It includes the processing time, waiting time, and transfer time of input and output data to and from the resources. Based on the TTR value, the tasks are arranged in descending order and scheduled to resources with minimum TTR. This algorithm performs better when compared to First Come First Serve (FCFS) scheduling and Min-min algorithms.

A divided Min-min scheduling algorithm [3] classifies jobs according to their ETC values as average, minimum, and maximum. Then, it divides the jobs into same size segments and schedules the large job segment first and then the small job segment. It uses Min-min algorithm for scheduling. Different from Min-min, it sorts jobs before scheduling, which means that the job with long execution time will be scheduled earlier.

A fault tolerance service based on different types of failures satisfying the QoS requirements is proposed in [4]. It has a fault detector, fault manager, resource manager, resource allocation manager, meta computing directory service, and execution time predictor. It allocates resources based on QoS requirements and performs job migration in case of occurrence of failures.

A Minimum Total Time to Release (MTTR) algorithm [5] reduces the time to release value by allocating computational resource based on job requirements, characteristics, and hardware features of resources. It adopts a check pointing based fault tolerance and the check points are based on failure rate. It proposes a Replica Resource Selection Algorithm to provide checkpoint replication service.

In [6], the root cause of failures is studied from the real time data and categorizes them as human, environment, network, software, and hardware. The failure rate is analyzed as a function of system and node and identified that the failure rates do not grow significantly faster than system size. Failure rate is analyzed at different time scales and statistical properties of time between failures are also defined.

The performance of most commonly used fault-tolerant techniques in grid computing is analyzed in [7]. The metrics such as throughput, turnaround time, waiting time, and network delay are considered for evaluation. The average percentage of faults and the workloads are varied to analyze the behavior of these techniques. It analyses the task level fault tolerance mechanisms such as retrying, alternate resource, check pointing, and replication.

The importance of fault tolerance for achieving reliability is surveyed [8] by all possible mechanisms such as replication, check pointing, and job migration. It extends the cost optimization algorithm to optimize the time without incurring additional processing expenses. This is accomplished by applying the time-optimization algorithm to schedule task farming or parameter-sweep application jobs on distributed resources having the same processing cost.

In [9], a fault tolerant scheduling architecture that employs job replication is proposed. The algorithm determines adaptively the number of job replicas based on resource failure history. Then, it schedules the replicas to efficient resources using the backup resource selection algorithm.

A cost optimization scheduling algorithm is described in [10] to optimize the cost to execute the jobs. It optimizes time, keeping the cost of computation at minimum. It also reduces the execution time of the jobs. But in this algorithm failure rate of the resources and user deadline of the jobs are not considered.

A static heuristic approach [11] is proposed for scheduling independent tasks in grid environment which considers user satisfaction. The requirements of tasks are necessary to identify suitable resources. The proposed scheduling algorithm considers both system and application aspects, that is, the factors to improve the system performance and utilization of the resources and throughput. It makes use of the user deadline of tasks, data transfer time, and the computation time for each 〈job, resource〉 pair for making scheduling decisions.

A grouping based scheduling algorithm [12] is proposed which considers user deadline and reduces communication time by adopting the grouping technique. The grouping strategy followed in this algorithm groups the fine grained tasks to coarse grained tasks based on the user deadline and computation time.

An efficient load balancing and grouping based job scheduling approach for grouping of fine-grained jobs is proposed in [13]. Its main goal is to maximize resource utilization and minimize processing time of tasks. It schedules tasks based on number of tasks available at a particular time and resource capability. Independent fine-grained jobs are grouped together based on the dynamically specified group size and resource characteristics.

A neighbour level load balancing mechanism is proposed in [14]. A more accurate load measurement method is applied to determine the load of each resource. A load balancing algorithm is executed based on the information exchanged between neighbour nodes. If any node is overloaded then the load on every neighbour's node is evaluated and finds underloaded nodes. Then the task is shifted to underloaded nodes.

A hybrid load balancing policy which integrates static and dynamic load balancing technologies is proposed in [15]. Essentially, a static load balancing policy is applied to select effective and suitable node sets. It reduces the unbalanced load probability caused by assigning tasks to ineffective nodes. When a node reveals the possible inability to continue providing resources, the dynamic load balancing policy will determine whether the node in question is ineffective to provide load assignment. The system will then obtain a new replacement node within a short time, to maintain system execution performance.

A system level load balancing [16] is proposed where a distributed load balancing model transforms grid topology into a forest structure. A two-level strategy is proposed to balance the load among resources of computational grid. In level 0, each cluster manager is associated with a physical cluster of the grid. The cluster manager is responsible for maintaining the workload information related to each one of its worker nodes, estimating the workload of associated cluster and diffusing this information to other cluster managers, deciding to start intracluster load balancing, sending the load balancing decisions to the worker nodes which they manage for execution and initiating the intercluster load balancing. In level 1, the worker nodes of a grid that are linked to their respective clusters are determined. Each node at this level is responsible for maintaining its workload information, sending this information to its cluster manager and performing the load balancing decided by its cluster manager. Load balancing schemes for grid environment [17] are proposed that do not follow the changes in the system status or set fixed threshold for controlling the load.

A dynamic and distributed protocol is designed in [18]. The grid is partitioned into a number of clusters. Each cluster has a coordinator to perform local load balancing decisions and also to communicate with other cluster coordinators across the grid to provide intercluster load transfers. The distributed protocol uses the clusters of the grid to perform local load balancing decision within the clusters and if this is not possible, load balancing is performed among the clusters under the control of cluster coordinators.

A fault tolerant hybrid load balancing algorithm [19] is proposed which is carried out in two phases: static load balancing and dynamic load balancing. In the first phase, a static load balancing policy selects the desired effective sites to carry out the submitted job. If any of the sites is unable to complete the assigned job, then a new site will be located using the dynamic load balancing policy. The assignment of jobs must be adjusted dynamically in accordance with the variation of site status. The variation in site status can be identified at any of the cases when the grid scheduler receives the message that a certain site can no longer provide resources or when job execution on a certain site exceeds the expected execution time or when the site is overloaded.

A load balancing mechanism, which works in 2 phases, is proposed in [20]. In the first phase, job allocation is done based on a defined criterion; that is, the heuristic begins with the set of all unmapped tasks. Then the set of minimum completion times is found, like Min-min heuristic. In second phase, heuristic algorithm works based on machines workload, which consists of two steps.

In the first step, for each task the first and second minimum completion time and the minimum execution time are found. Then the difference between these two minimum completion time values is multiplied by the amount of minimum completion time and then divided by minimum execution time. In the second step, if the number of the remaining tasks is not less than threshold, then the heuristic algorithm is executed to balance the load. Finally, the task which has the criteria value as maximum will be selected and removed from the set of unmapped tasks.

A dynamic, distributed load balancing scheme for a grid environment is proposed [21] which provides deadline control for tasks. Periodically the resources check their state and make a request to the grid broker according to the change of state in load. Then, the grid broker assigns gridlets based on deadline request and load. In [22], a hybrid algorithm is proposed for optimal load sharing with two components such as hash table and distributed hash table. It finds the nearest node and shares the load of a highly loaded node to lightly loaded node. It proves to provide the best tradeoff between space usage and lookup time. All these algorithms mentioned in literature concentrate on load balancing, fault tolerance, and user satisfaction to an extent. But none of them considers all these factors combined. This research proposes a Multiconstrained Load Balancing Fault Tolerant Algorithm (MLFT) which considers all these factors during scheduling. The architecture and the algorithm of MLFT are explained below. In our previous work [23], we have proposed a new Bicriteria Scheduling Algorithm that considers both user satisfaction and fault tolerance. The proactive fault tolerant technique is adopted and the scheduling is carried out by considering the deadline of gridlets submitted. The main contribution of this paper includes achieving user satisfaction along with fault tolerance and minimizing the makespan of jobs. In our previous work [24], we have proposed a multicriteria scheduling algorithm that considers load balancing, fault tolerance, and user satisfaction as a centralized approach.

In our previous work [25], we have proposed an efficient fault tolerant scheduling algorithm (FTMM) which is based on data transfer time and failure rate. System performance is also achieved by reducing the idle time of the resources and distributing the unmapped tasks equally among the available resources.

A prioritized user demand algorithm is proposed [26] that considers user deadline for allocating jobs to different heterogeneous resources from different administrative domains. It produces better makespan and more user satisfaction but data requirement is not considered. While scheduling the jobs, failure rate is not considered. So the scheduled jobs may fail during execution.

A work based on user satisfaction and hierarchical load balancing is proposed [27] that considers user demands and load balancing. It minimizes the response time of the jobs and improves the utilization of the resources in grid environment. By considering the user demand of the jobs, the scheduling algorithm also improves the user satisfaction.

With this study, an algorithm is proposed which is centralized and considers cost as a scheduling parameter in addition to the previously proposed scheduling parameters.

## 2. Materials and Methods

### 2.1. Problem Formulation

**Figure 1:**
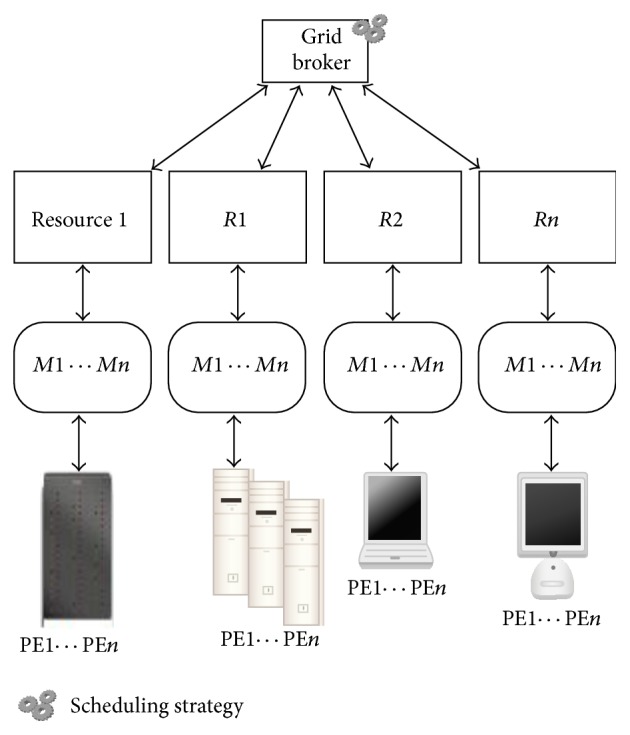
Centralized scheduling architecture.

The proposed algorithm follows a centralized scheduling architecture depicted in Figure 1 where the scheduling is done only at the grid broker. Also it follows a static batch mode scheduling in which the tasks are scheduled in batches and when a task is allocated with a resource, it will not be changed. Hence the proposed algorithm is static, batch mode, and centralized scheduling algorithm.

### 2.2. Proposed MLFT Scheduling Architecture

**Figure 2:**
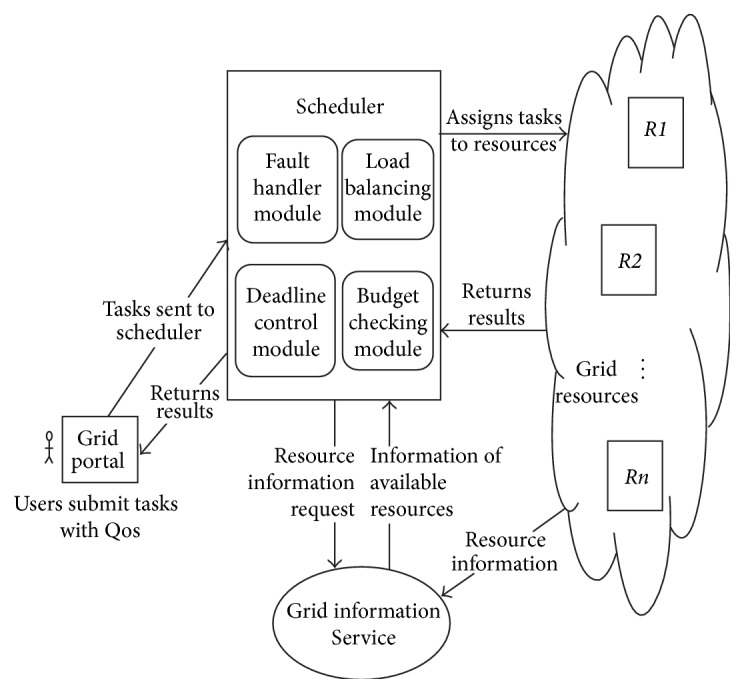
MLFT architecture.

The scheduling architecture MLFT algorithm is depicted in Figure 2. The users submit the tasks to the grid broker through grid portal. The tasks are submitted along with the QoS requirements such as task completion deadline and execution cost. The grid portal submits the tasks to the grid scheduler/broker. The architecture has a Grid Information Service (GIS) which collects the information of all the resources involved in grid such as initial failure rate, number of tasks submitted, number of tasks successfully completed, availability time, and processing capability in MIPS. The scheduler has four components.

**Figure 3:**
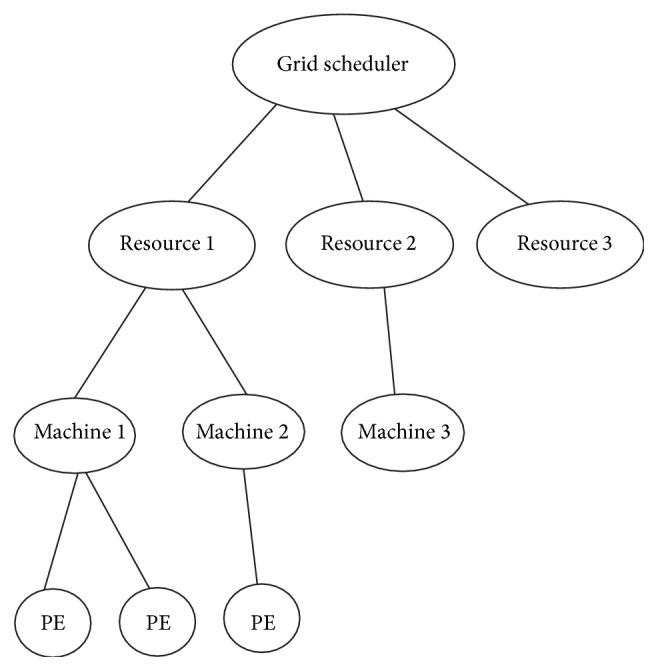
Gridsim architecture.

The first is the fault handler module which calculates the failure rate of each resource and checks whether the selected resource has less failure rate. The second component is the deadline control module which takes care of user satisfaction in terms of satisfied deadline for task completion. The third is the load balancing module which updates the load of each resource and keeps control of balanced load. The fourth scheduler component is the budget control module which ensures minimized execution cost. This algorithm is implemented using the GridSim which follows the architecture depicted in Figure 3.

### 2.3. Proposed MLFT Algorithm

The proposed MLFT algorithm follows a static batch mode scheduling strategy in a centralized fashion. The algorithm is implemented at the grid broker level. It is given in Algorithm 1 and it works as follows.

At the time of task submission to grid portal, the user submits the deadline UD(*T*_*i*_) and budget *B*(*T*_*i*_) for task completion. The GIS receives the information of all the resources involved in grid such as computation cost CS(*R*_*j*_). The algorithm makes use of these resource information and the user requirements and performs scheduling.

The load balancing module performs calculation of load and threshold value at all levels as follows.

The load of each processing element is calculated by using the weighted sum of squares which is given by

(1) $$\begin{array}{c} Load\left(\;{PE}_{i}\;\right)=\sqrt{\overset{n}{\underset{k=1}{\sum }}\left({{a_{k}L}_{k}}^{2}\right)}, \end{array}$$

where *L*_*k*_ is the load attribute considered in our algorithm [24]. The load attribute considered in our algorithm is the CPU utilization in seconds. The value of *a*_*k*_ which is the weight of each attribute is considered as 1 and hence the load of PE is given by

(2) $$\begin{array}{c} Load\left(\;{PE}_{i}\;\right)=\frac{\sum_{j=0}^{n}{MI}_{j}}{{MIPS}_{i}}, \end{array}$$

where *n* is the number of tasks allocated to PE_*i*_. The average load of each machine is calculated with the loads of PE's such as

(3) $$\begin{array}{c} AL\left(\;M_{i}\;\right)=\frac{\sum_{k=1}^{n}Load\left({PE}_{k}\right)}{PE\_num}, \end{array}$$

where PE_num is the number of PE's under Machine *i*. The average load of each resource is calculated by

(4) $$\begin{array}{c} AL\left(\;R_{i}\;\right)=\frac{\sum_{k=1}^{n}AL\left(M_{k}\right)}{M\_num}, \end{array}$$

where *M*_num is the number of machines under resource *i*.

The average load of the system/grid broker is calculated as

(5) $$\begin{array}{c} AL=\frac{\sum_{k=1}^{n}AL\left(R_{k}\right)}{R\_num}, \end{array}$$

where *R*_num is the number of resources in the system. After calculating the load of the resources, threshold value at the grid broker level is calculated as

(6) $$\begin{array}{c} Ω=AL+\sigma , \end{array}$$

where

(7) $$\begin{array}{c} \sigma =\sqrt{\frac{\sum_{i=1}^{N}{\left(AL\left(R_{i}\right)-AL\right)}^{2}}{N}}, \end{array}$$

where *N* is the number of resources in the system. In terms of gridsim the tasks are represented as gridlets.

The information of the gridlet such as gridlet size in million instructions (MI) is used to calculate the Expected Time to Compute matrix for all gridlets in all resources by using the formula

(8) $$\begin{array}{c} ETC\left(\;T_{i},R_{j}\;\right)=\frac{{Length}_{i}}{{Capacity}_{j}}. \end{array}$$

The completion time matrix is calculated for each gridlet in each resource as

(9) $$\begin{array}{c} CT\left(\;T_{i},R_{j}\;\right)=ETC\left(\;T_{i},R_{j}\;\right)+RT\left(\;R_{j}\;\right) \end{array}$$

and the total completion time is calculated as

(10) $$\begin{array}{c} TCT\left(\;T_{i},R_{j}\;\right)=CT\left(\;T_{i},R_{j}\;\right)+CMT\left(\;T_{i},R_{j}\;\right). \end{array}$$

The budget control module calculates the cost matrix CST(*T*_*i*_, *R*_*j*_) for executing each gridlet in each resource as

(11) $$\begin{array}{c} CST\left(\;T_{i},R_{j}\;\right)=ETC\left(\;T_{i},R_{j}\;\right)\times CS\left(\;R_{j}\;\right). \end{array}$$

The cost of execution and the expected budget from the user are compared and a suitable resource is selected.

The fault handler module calculates the failure rate of each resource with the information such as number of gridlets submitted and successfully completed. It is calculated using the formula

(12) $$\begin{array}{c} FR\left(\;R_{j}\;\right)=\frac{T_{f}}{T_{sub}}, \end{array}$$

where *T*_*f*_  is the number of tasks failed to be executed previously in resource *j* and *T*_sub_ is the number of tasks submitted to resource *j* for execution. The ready time of each resource is calculated by

(13) $$\begin{array}{c} RT\left(\;R_{j}\;\right)=\overset{n}{\underset{i=1}{\sum }}ETC\left(\;T_{i},R_{j}\;\right), \end{array}$$

where *n* is the number of tasks submitted to *R*_*j*_.

## 3. Results and Discussion

### 3.1. Experimental Setup

The proposed algorithm aims at reducing the makespan and scheduling efficiently with fault tolerance and balanced load. Also, the user satisfaction is considered with deadline control and budget parameters. The fault tolerance is ensured with improved hit rate and the user satisfaction is ensured with increased deadline hit count and reduced processing/execution cost. The balanced load is ensured with highest average resource utilization. Gridsim 5.0 toolkit is used for evaluating the proposed algorithm based on these factors:

 Number of resources: 16.
 Number of tasks: 512.

The gridlets assumed are independent and computationally intensive and arrive randomly and follow Poisson process. It is assumed that each resource can execute a single gridlet at a time. The resource characteristics and the parameters considered for scheduling are given in Tables 1 and 2, respectively. The task and machine heterogeneity is considered for comparison. Four categories of data such as High Task Low Machine, Low Task High Machine, High Task High Machine, and Low Task Low Machine are considered.

### 3.2. Performance Metrics

The proposed algorithm is designed to satisfy the user with respect to deadline and budget, balanced load, and fault tolerance. The performance metrics used to evaluate the proposed MLFT algorithm are makespan, hit count, deadline hit count, average resource utilization, and execution cost. These performance metrics are defined below.

*Makespan*. This metric is for evaluating the overall performance of the scheduling algorithm. It is defined as the overall completion time of a batch of tasks and is given by

(14) $$\begin{array}{c} Makespan=\max\left\{\;RT\left(\;R_{j}\;\right)\;\right\},\;\forall j\in n. \end{array}$$

It is used to measure the ability of grid to accommodate gridlets in less time. 

*Hit Count*. Hit count is a new metric introduced that represents the number of tasks successfully completed in a batch of tasks. Here, each batch is assumed to have 512 tasks and the hit count gives the number of tasks successfully completed out of 512. 

*Deadline Hit Count*. This is a new metric introduced which represents the number of tasks successfully completed within the given user deadline. 

*Average Resource Utilization*. This metric is newly introduced in order to measure the load balancing which can be calculated as follows. The utilization of each resource RU(*R*_*j*_) can be calculated by

(15) $$\begin{array}{c} RU\left(\;R_{j}\;\right)=\frac{\sum_{i=0}^{m}{MI}_{i}}{{MIPS}_{j}\times {AT}_{j}}\times 100. \end{array}$$

The average resource utilization ARU of the system can be calculated using

(16) $$\begin{array}{c} ARU=\frac{1}{N}\overset{N}{\underset{j=1}{\sum }}RU\left(\;R_{j}\;\right), \end{array}$$

where *N* is the number of resources. 

*Processing Cost*. This metric is newly introduced in order to measure the algorithm's performance based on user satisfaction based on budget.

### 3.3. Experimental Results

The proposed MLFT algorithm is compared with the Min-min algorithm which stands as a benchmark static heuristic algorithm for grid scheduling and the Fault Tolerant Algorithm (FTMM) proposed in [25], Bicriteria Scheduling Algorithm (BSA) [23], and LBFT algorithm [24] which is a load balancing algorithm for proving its performance based on makespan, hit count, deadline hit count, average resource utilization, and cost.

**Figure 4:**
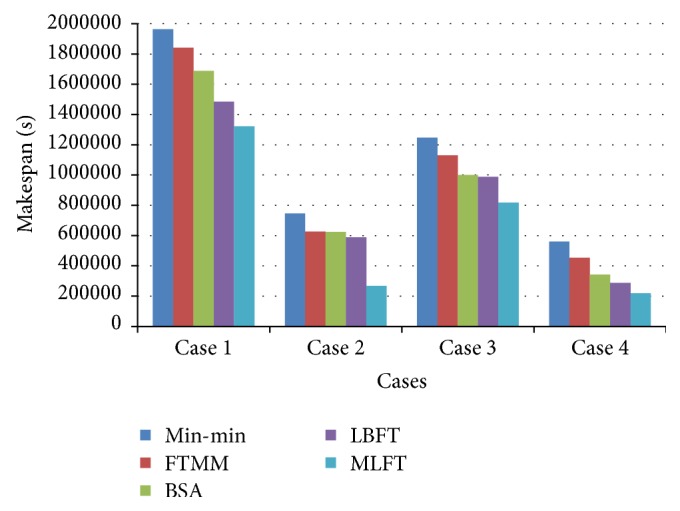
Performance based on makespan (sec).

The performance comparison of the proposed MLFT algorithm based on makespan is shown in Figure 4. The results show that the MLFT has minimized makespan than the other algorithms.

**Figure 5:**
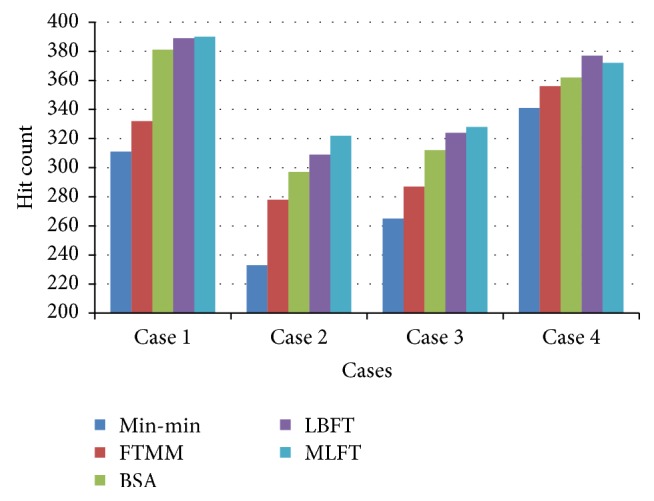
Performance based on hit count.

The performance of MLFT based on hit count which is the measure of fault tolerance is shown in Figure 5. The results show that the MLFT algorithm has more number of gridlets successfully completed without failure.

**Figure 6:**
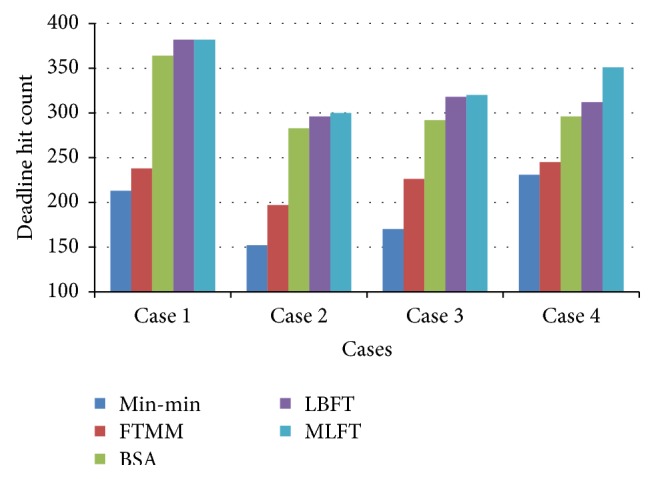
Performance based on deadline hit count.

The results of MLFT based on deadline hit count are shown in Figure 6. It is inferred that when compared with other algorithms such as Min-min, FTMM, BSA, and LBFT, the proposed MLFT has increased number of gridlets completed within user deadline.

**Figure 7:**
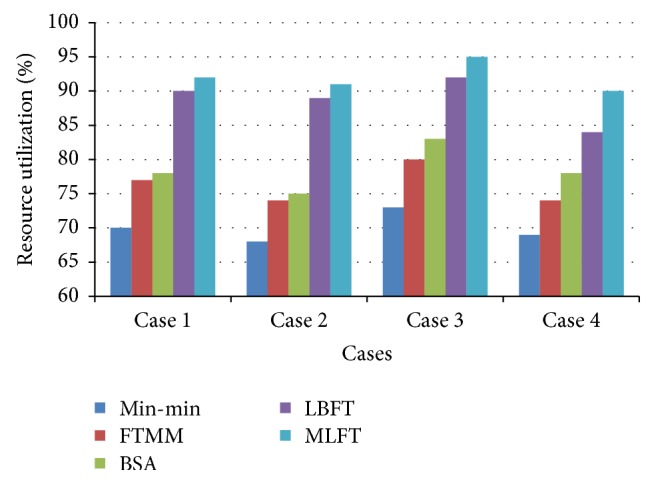
Performance based on resource utilization (%).

The results based on resource utilization is shown in Figure 7 and it is inferred that the proposed MLFT algorithm has better resource utilization than the other algorithms such as Min-min, FTMM, BSA, and LBFT which concentrates separately on each factor.

**Figure 8:**
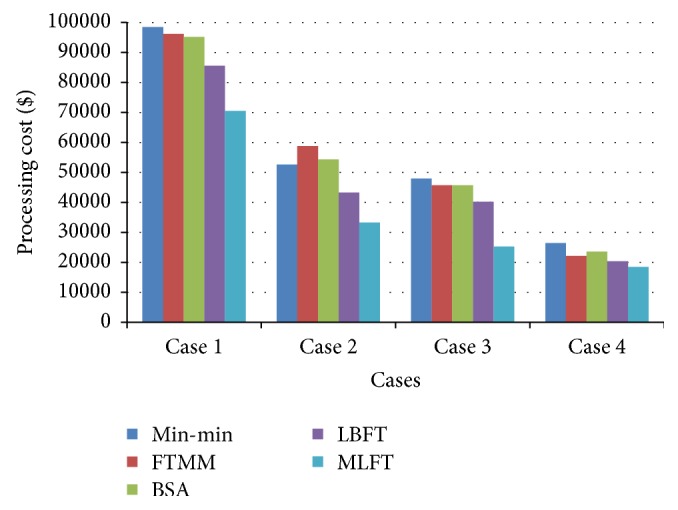
Performance based on processing cost.

The performance of MLFT based on processing cost is shown in Figure 8. The cost required to execute a batch of tasks is comparatively less for MLFT than Min-min, FTMM, BSA, and LBFT algorithms which do not concentrate on processing cost.

## 4. Conclusions and Future Work

In this work, a budget constrained scheduling algorithm which mainly concentrates on processing cost is proposed. By reducing the processing cost, it makes an attempt to satisfy the user. Along with this cost factor, it also considers user deadline of task completion to satisfy the user. With these two factors considered for user satisfaction, it also takes care of proper resource utilization and fault tolerance with reduced makespan.

The efficiency of this algorithm is proved by comparing it with already existing algorithms which separately concentrates on these factors based on makespan, hit count, deadline hit count, resource utilization, and processing cost. The applications considered in this work are computation intensive. In the future, this can be extended for data intensive applications. This algorithm follows a centralized approach and in the future, this can be extended in a hierarchical environment.

## Figures and Tables

**Algorithm 1:**
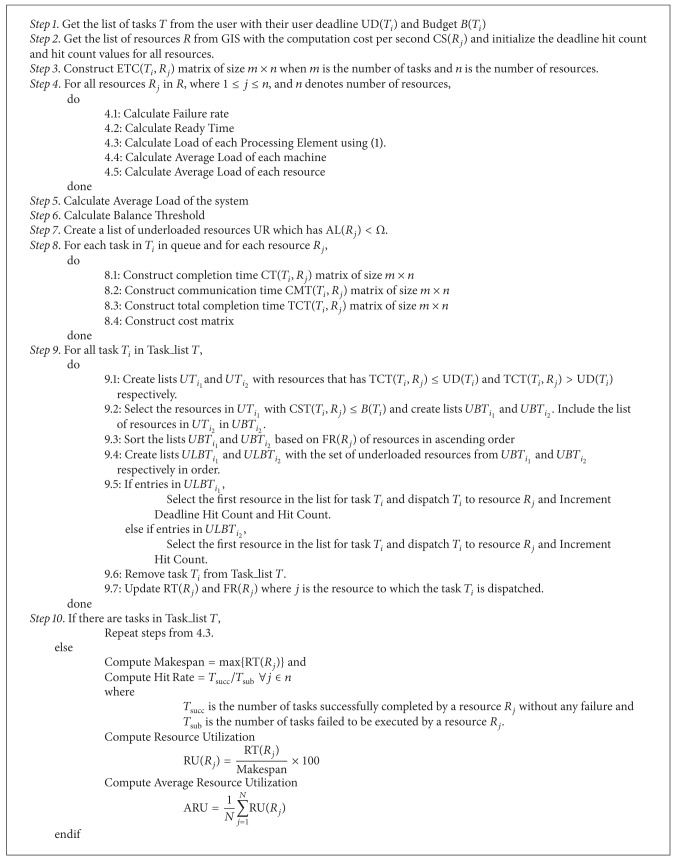
MLFT scheduling algorithm.

**Table 1:** Grid resource characteristics.

Number of machines	1–4

Number of PE's per machine	1-2

PE ratings	5 to 50 MIPS

**Table 2:** Scheduling parameters and their values.

Number of gridlets	512

Gridlet length (MI)	50,000 to 1,00,000

I/P file size	50 to 500 MB

O/P file size	100 to 700 MB
